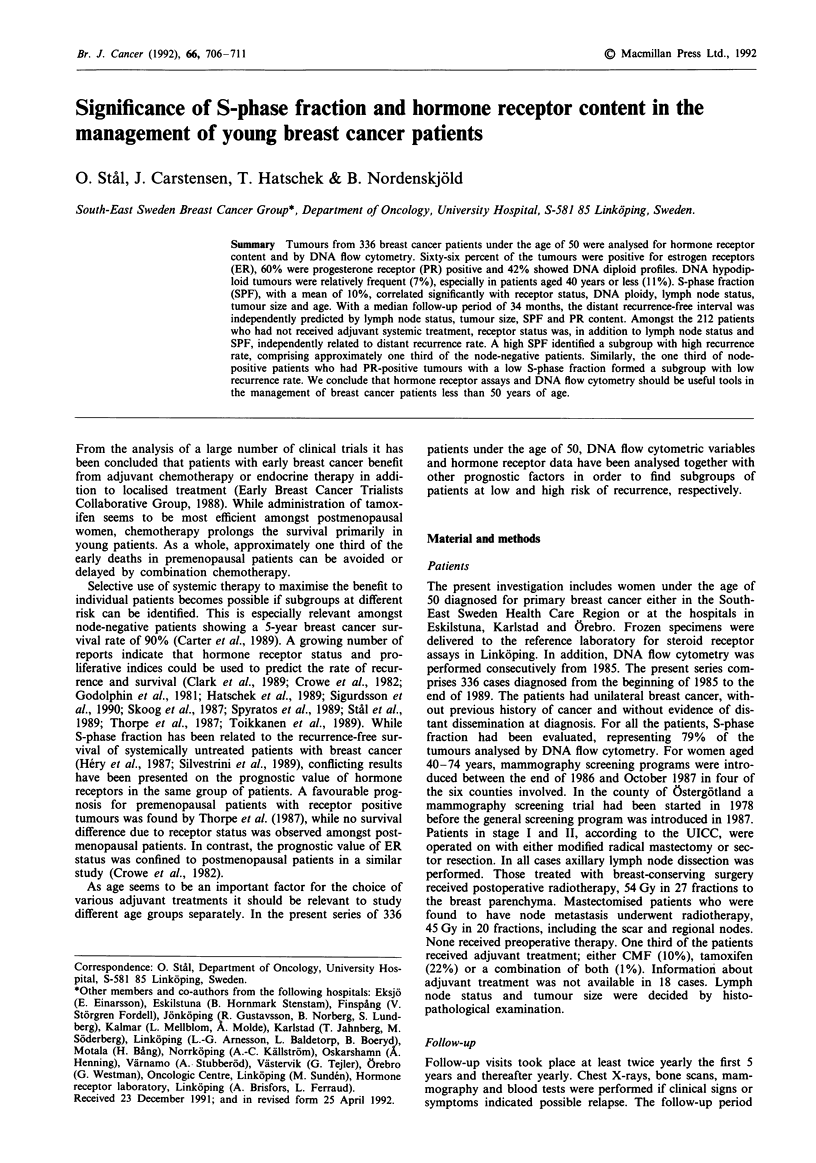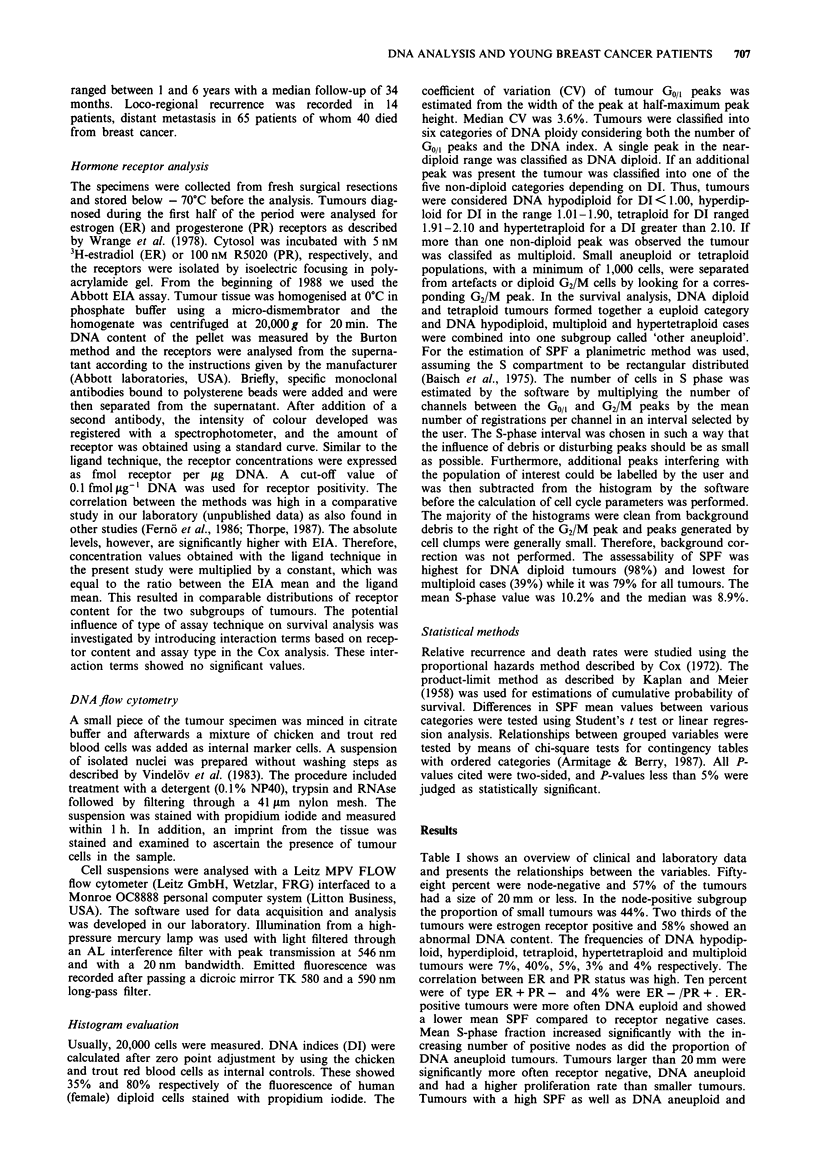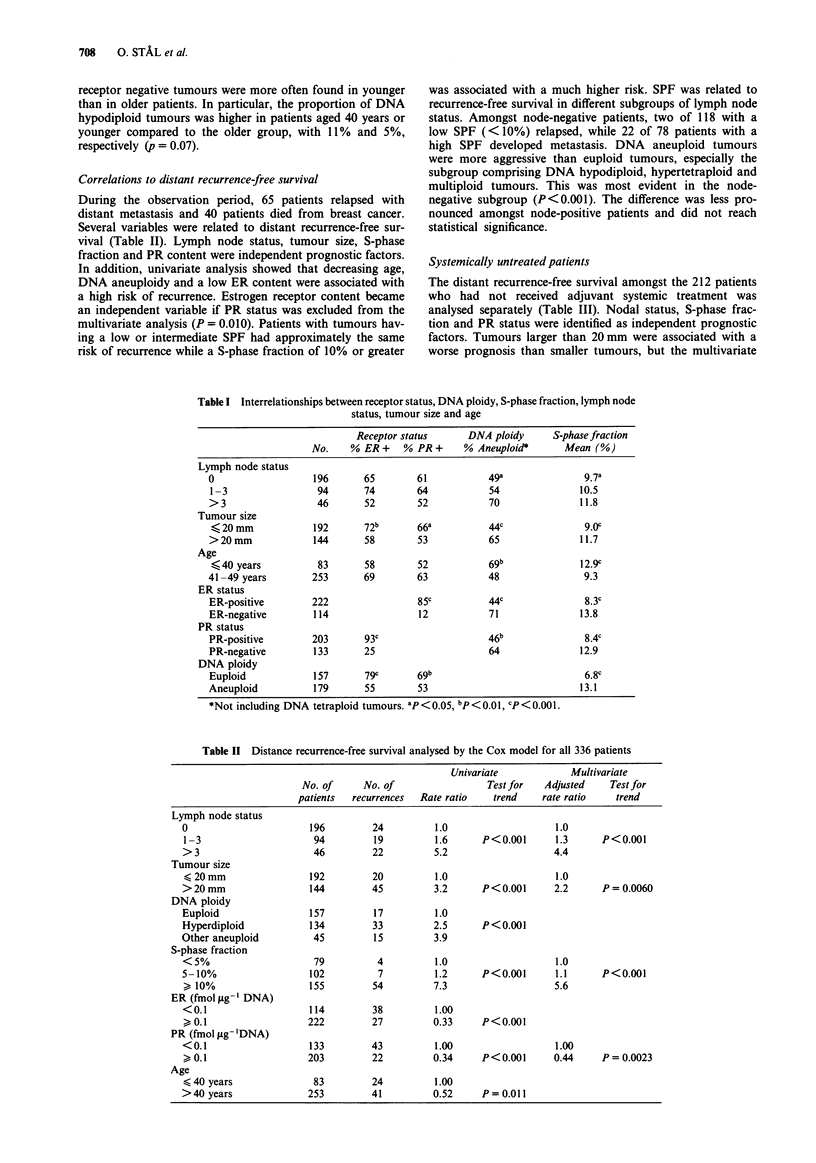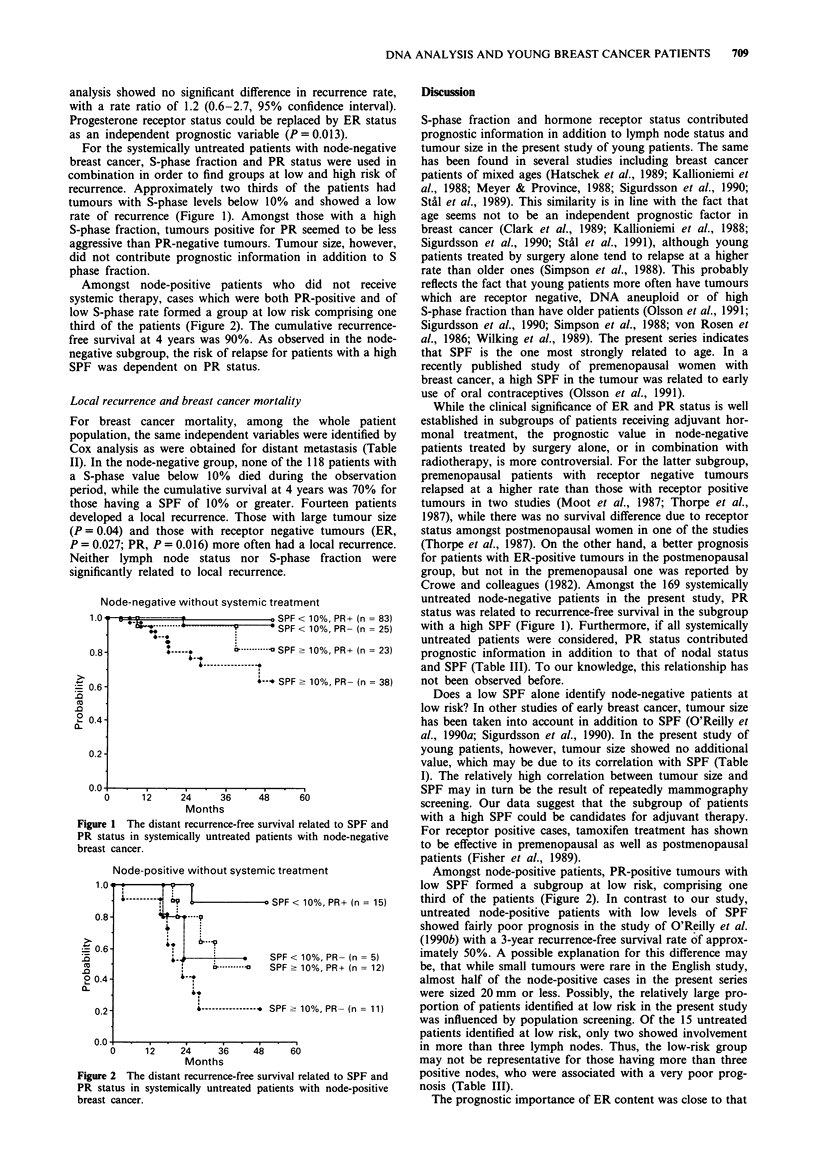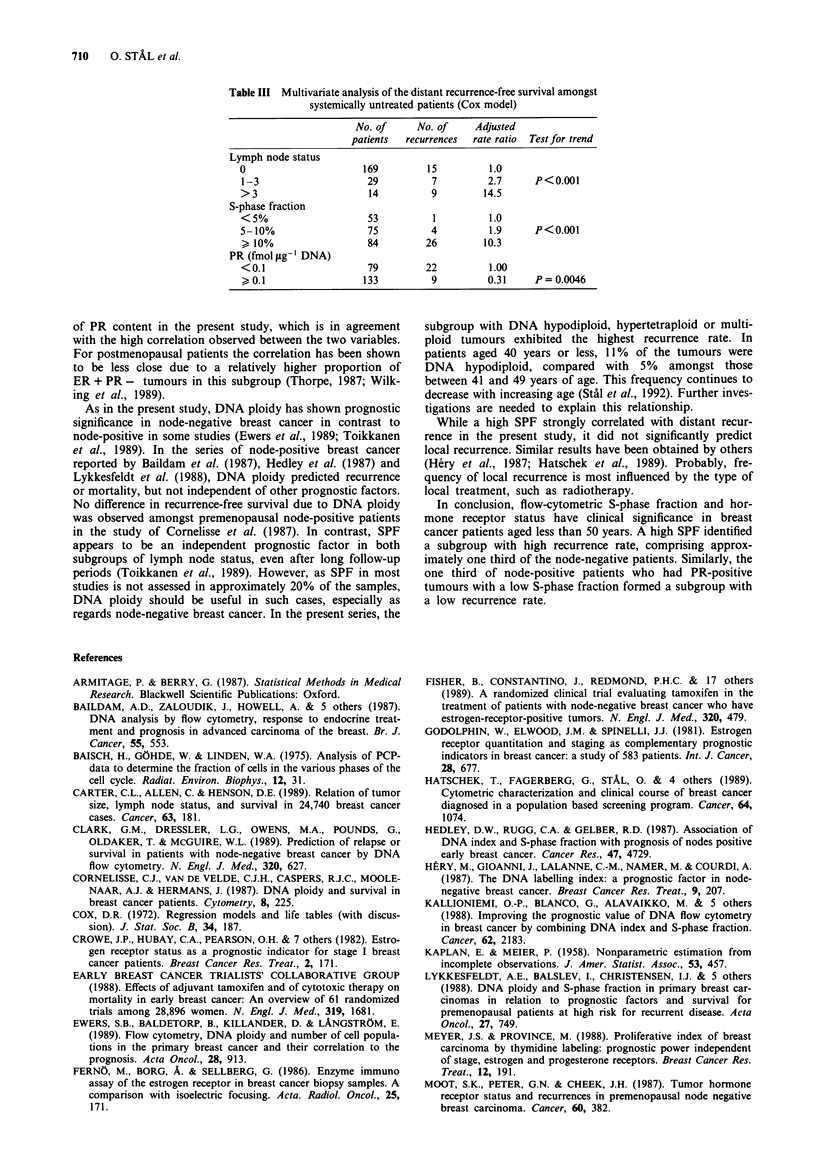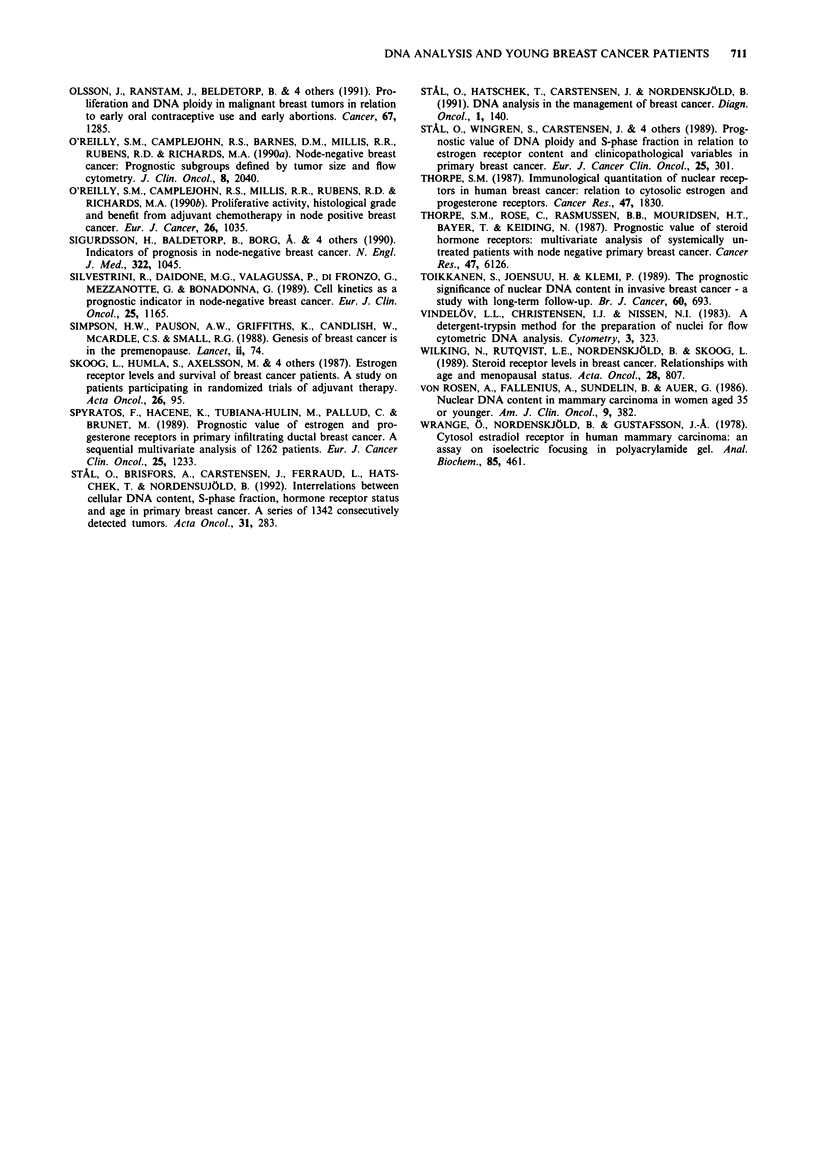# Significance of S-phase fraction and hormone receptor content in the management of young breast cancer patients.

**DOI:** 10.1038/bjc.1992.342

**Published:** 1992-10

**Authors:** O. Stål, J. Carstensen, T. Hatschek, B. Nordenskjöld

**Affiliations:** Department of Oncology, University Hospital, Linköping, Sweden.

## Abstract

Tumours from 336 breast cancer patients under the age of 50 were analysed for hormone receptor content and by DNA flow cytometry. Sixty-six percent of the tumours were positive for estrogen receptors (ER), 60% were progesterone receptor (PR) positive and 42% showed DNA diploid profiles. DNA hypodiploid tumours were relatively frequent (7%), especially in patients aged 40 years or less (11%). S-phase fraction (SPF), with a mean of 10%, correlated significantly with receptor status, DNA ploidy, lymph node status, tumour size and age. With a median follow-up period of 34 months, the distant recurrence-free interval was independently predicted by lymph node status, tumour size, SPF and PR content. Amongst the 212 patients who had not received adjuvant systemic treatment, receptor status was, in addition to lymph node status and SPF, independently related to distant recurrence rate. A high SPF identified a subgroup with high recurrence rate, comprising approximately one third of the node-negative patients. Similarly, the one third of node-positive patients who had PR-positive tumours with a low S-phase fraction formed a subgroup with low recurrence rate. We conclude that hormone receptor assays and DNA flow cytometry should be useful tools in the management of breast cancer patients less than 50 years of age.


Br. J. Cancer (1992), 66, 706–711 © Macmillan Press Ltd., 1992

### Significance of S-phase fraction and hormone receptor content in the management of young breast cancer patients

O. Stål, J. Carstensen, T. Hatschek & B. Nordenskjöld

*South-East Sweden Breast Cancer Group*, Department of Oncology, University Hospital, S-581 85 Linköping, Sweden.*

**Summary** Tumours from 336 breast cancer patients under the age of 50 were analysed for hormone receptor content and by DNA flow cytometry. Sixty-six percent of the tumours were positive for estrogen receptors (ER), 60% were progesterone receptor (PR) positive and 42% showed DNA diploid profiles. DNA hypodiploid tumours were relatively frequent (7%), especially in patients aged 40 years or less (11%). S-phase fraction (SPF), with a mean of 10%, correlated significantly with receptor status, DNA ploidy, lymph node status, tumour size and age. With a median follow-up period of 34 months, the distant recurrence-free interval was independently predicted by lymph node status, tumour size, SPF and PR content. Amongst the 212 patients who had not received adjuvant systemic treatment, receptor status was, in addition to lymph node status and SPF, independently related to distant recurrence rate. A high SPF identified a subgroup with high recurrence rate, comprising approximately one third of the node-negative patients. Similarly, the one third of node-positive patients who had PR-positive tumours with a low S-phase fraction formed a subgroup with low recurrence rate. We conclude that hormone receptor assays and DNA flow cytometry should be useful tools in the management of breast cancer patients less than 50 years of age.

From the analysis of a large number of clinical trials it has been concluded that patients with early breast cancer benefit from adjuvant chemotherapy or endocrine therapy in addition to localised treatment (Early Breast Cancer Trialists Collaborative Group, 1988). While administration of tamoxifen seems to be most efficient amongst postmenopausal women, chemotherapy prolongs the survival primarily in young patients. As a whole, approximately one third of the early deaths in premenopausal patients can be avoided or delayed by combination chemotherapy.

Selective use of systemic therapy to maximise the benefit to individual patients becomes possible if subgroups at different risk can be identified. This is especially relevant amongst node-negative patients showing a 5-year breast cancer survival rate of 90% (Carter *et al.*, 1989). A growing number of reports indicate that hormone receptor status and proliferative indices could be used to predict the rate of recurrence and survival (Clark *et al.*, 1989; Crowe *et al.*, 1982; Godolphin *et al.*, 1981; Hatschek *et al.*, 1989; Sigurdsson *et al.*, 1990; Skoog *et al.*, 1987; Spyratos *et al.*, 1989; Stål *et al.*, 1989; Thorpe *et al.*, 1987; Toikkanen *et al.*, 1989). While S-phase fraction has been related to the recurrence-free survival of systemically untreated patients with breast cancer (Héry *et al.*, 1987; Silvestrini *et al.*, 1989), conflicting results have been presented on the prognostic value of hormone receptors in the same group of patients. A favourable prognosis for premenopausal patients with receptor positive tumours was found by Thorpe *et al.* (1987), while no survival difference due to receptor status was observed amongst postmenopausal patients. In contrast, the prognostic value of ER status was confined to postmenopausal patients in a similar study (Crowe *et al.*, 1982).

As age seems to be an important factor for the choice of various adjuvant treatments it should be relevant to study different age groups separately. In the present series of 336 patients under the age of 50, DNA flow cytometric variables and hormone receptor data have been analysed together with other prognostic factors in order to find subgroups of patients at low and high risk of recurrence, respectively.

Correspondence: O. Stål, Department of Oncology, University Hospital, S-581 85 Linköping, Sweden.

*Other members and co-authors from the following hospitals: Eksjö (E. Einarsson), Eskilstuna (B. Hornmark Stenstam), Finspång (V. Störgren Fordell), Jönköping (R. Gustavsson, B. Norberg, S. Lundberg), Kalmar (L. Mellblom, Å. Molde), Karlstad (T. Jahnberg, M. Söderberg), Linköping (L.-G. Arnesson, L. Baldetorp, B. Boeryd), Motala (H. Bång), Norrköping (A.-C. Källström), Oskarshamn (Å. Henning), Värnamo (A. Stubberöd), Västervik (G. Tejler), Örebro (G. Westman), Oncologic Centre, Linköping (M. Sundén), Hormone receptor laboratory, Linköping (A. Brisfors, L. Ferraud).

Received 23 December 1991; and in revised form 25 April 1992.

### Material and methods

### *Patients*

The present investigation includes women under the age of 50 diagnosed for primary breast cancer either in the South-East Sweden Health Care Region or at the hospitals in Eskilstuna, Karlstad and Örebro. Frozen specimens were delivered to the reference laboratory for steroid receptor assays in Linköping. In addition, DNA flow cytometry was performed consecutively from 1985. The present series comprises 336 cases diagnosed from the beginning of 1985 to the end of 1989. The patients had unilateral breast cancer, without previous history of cancer and without evidence of distant dissemination at diagnosis. For all the patients, S-phase fraction had been evaluated, representing 79% of the tumours analysed by DNA flow cytometry. For women aged 40–74 years, mammography screening programs were introduced between the end of 1986 and October 1987 in four of the six counties involved. In the county of Östergötland a mammography screening trial had been started in 1978 before the general screening program was introduced in 1987. Patients in stage I and II, according to the UICC, were operated on with either modified radical mastectomy or sector resection. In all cases axillary lymph node dissection was performed. Those treated with breast-conserving surgery received postoperative radiotherapy, 54 Gy in 27 fractions to the breast parenchyma. Mastectomised patients who were found to have node metastasis underwent radiotherapy, 45 Gy in 20 fractions, including the scar and regional nodes. None received preoperative therapy. One third of the patients received adjuvant treatment; either CMF (10%), tamoxifen (22%) or a combination of both (1%). Information about adjuvant treatment was not available in 18 cases. Lymph node status and tumour size were decided by histopathological examination.

### *Follow-up*

Follow-up visits took place at least twice yearly the first 5 years and thereafter yearly. Chest X-rays, bone scans, mammography and blood tests were performed if clinical signs or symptoms indicated possible relapse. The follow-up period ranged between 1 and 6 years with a median follow-up of 34 months. Loco-regional recurrence was recorded in 14 patients, distant metastasis in 65 patients of whom 40 died from breast cancer.

*Hormone receptor analysis*

The specimens were collected from fresh surgical resections and stored below − 70°C before the analysis. Tumours diagnosed during the first half of the period were analysed for estrogen (ER) and progesterone (PR) receptors as described by Wrange *et al.* (1978). Cytosol was incubated with 5 nM $^3$H-estradiol (ER) or 100 nM R5020 (PR), respectively, and the receptors were isolated by isoelectric focusing in polyacrylamide gel. From the beginning of 1988 we used the Abbott EIA assay. Tumour tissue was homogenised at 0°C in phosphate buffer using a micro-dismembrator and the homogenate was centrifuged at 20,000 *g* for 20 min. The DNA content of the pellet was measured by the Burton method and the receptors were analysed from the supernatant according to the instructions given by the manufacturer (Abbott laboratories, USA). Briefly, specific monoclonal antibodies bound to polysterene beads were added and were then separated from the supernatant. After addition of a second antibody, the intensity of colour developed was registered with a spectrophotometer, and the amount of receptor was obtained using a standard curve. Similar to the ligand technique, the receptor concentrations were expressed as fmol receptor per μg DNA. A cut-off value of 0.1 fmol $\mu g^{-1}$ DNA was used for receptor positivity. The correlation between the methods was high in a comparative study in our laboratory (unpublished data) as also found in other studies (Fernö *et al.*, 1986; Thorpe, 1987). The absolute levels, however, are significantly higher with EIA. Therefore, concentration values obtained with the ligand technique in the present study were multiplied by a constant, which was equal to the ratio between the EIA mean and the ligand mean. This resulted in comparable distributions of receptor content for the two subgroups of tumours. The potential influence of type of assay technique on survival analysis was investigated by introducing interaction terms based on receptor content and assay type in the Cox analysis. These interaction terms showed no significant values.

*DNA flow cytometry*

A small piece of the tumour specimen was minced in citrate buffer and afterwards a mixture of chicken and trout red blood cells was added as internal marker cells. A suspension of isolated nuclei was prepared without washing steps as described by Vindelöv *et al.* (1983). The procedure included treatment with a detergent (0.1% NP40), trypsin and RNAse followed by filtering through a 41 μm nylon mesh. The suspension was stained with propidium iodide and measured within 1 h. In addition, an imprint from the tissue was stained and examined to ascertain the presence of tumour cells in the sample.

Cell suspensions were analysed with a Leitz MPV FLOW flow cytometer (Leitz GmbH, Wetzlar, FRG) interfaced to a Monroe OC8888 personal computer system (Litton Business, USA). The software used for data acquisition and analysis was developed in our laboratory. Illumination from a high-pressure mercury lamp was used with light filtered through an AL interference filter with peak transmission at 546 nm and with a 20 nm bandwidth. Emitted fluorescence was recorded after passing a dicroic mirror TK 580 and a 590 nm long-pass filter.

*Histogram evaluation*

Usually, 20,000 cells were measured. DNA indices (DI) were calculated after zero point adjustment by using the chicken and trout red blood cells as internal controls. These showed 35% and 80% respectively of the fluorescence of human (female) diploid cells stained with propidium iodide. The coefficient of variation (CV) of tumour $G_{0/1}$ peaks was estimated from the width of the peak at half-maximum peak height. Median CV was 3.6%. Tumours were classified into six categories of DNA ploidy considering both the number of $G_{0/1}$ peaks and the DNA index. A single peak in the near-diploid range was classified as DNA diploid. If an additional peak was present the tumour was classified into one of the five non-diploid categories depending on DI. Thus, tumours were considered DNA hypodiploid for $DI < 1.00$, hyperdiploid for DI in the range 1.01–1.90, tetraploid for DI ranged 1.91–2.10 and hypertetraploid for a DI greater than 2.10. If more than one non-diploid peak was observed the tumour was classifed as multiploid. Small aneuploid or tetraploid populations, with a minimum of 1,000 cells, were separated from artefacts or diploid $G_2/M$ cells by looking for a corresponding $G_2/M$ peak. In the survival analysis, DNA diploid and tetraploid tumours formed together a euploid category and DNA hypodiploid, multiploid and hypertetraploid cases were combined into one subgroup called 'other aneuploid'. For the estimation of SPF a planimetric method was used, assuming the S compartment to be rectangular distributed (Baisch *et al.*, 1975). The number of cells in S phase was estimated by the software by multiplying the number of channels between the $G_{0/1}$ and $G_2/M$ peaks by the mean number of registrations per channel in an interval selected by the user. The S-phase interval was chosen in such a way that the influence of debris or disturbing peaks should be as small as possible. Furthermore, additional peaks interfering with the population of interest could be labelled by the user and was then subtracted from the histogram by the software before the calculation of cell cycle parameters was performed. The majority of the histograms were clean from background debris to the right of the $G_2/M$ peak and peaks generated by cell clumps were generally small. Therefore, background correction was not performed. The assessability of SPF was highest for DNA diploid tumours (98%) and lowest for multiploid cases (39%) while it was 79% for all tumours. The mean S-phase value was 10.2% and the median was 8.9%.

*Statistical methods*

Relative recurrence and death rates were studied using the proportional hazards method described by Cox (1972). The product-limit method as described by Kaplan and Meier (1958) was used for estimations of cumulative probability of survival. Differences in SPF mean values between various categories were tested using Student's *t* test or linear regression analysis. Relationships between grouped variables were tested by means of chi-square tests for contingency tables with ordered categories (Armitage & Berry, 1987). All *P*-values cited were two-sided, and *P*-values less than 5% were judged as statistically significant.

### Results

Table I shows an overview of clinical and laboratory data and presents the relationships between the variables. Fifty-eight percent were node-negative and 57% of the tumours had a size of 20 mm or less. In the node-positive subgroup the proportion of small tumours was 44%. Two thirds of the tumours were estrogen receptor positive and 58% showed an abnormal DNA content. The frequencies of DNA hypodiploid, hyperdiploid, tetraploid, hypertetraploid and multiploid tumours were 7%, 40%, 5%, 3% and 4% respectively. The correlation between ER and PR status was high. Ten percent were of type ER + PR − and 4% were ER − /PR + . ER-positive tumours were more often DNA euploid and showed a lower mean SPF compared to receptor negative cases. Mean S-phase fraction increased significantly with the increasing number of positive nodes as did the proportion of DNA aneuploid tumours. Tumours larger than 20 mm were significantly more often receptor negative, DNA aneuploid and had a higher proliferation rate than smaller tumours. Tumours with a high SPF as well as DNA aneuploid and receptor negative tumours were more often found in younger than in older patients. In particular, the proportion of DNA hypodiploid tumours was higher in patients aged 40 years or younger compared to the older group, with 11% and 5%, respectively ($p = 0.07$).

### *Correlations to distant recurrence-free survival*

During the observation period, 65 patients relapsed with distant metastasis and 40 patients died from breast cancer. Several variables were related to distant recurrence-free survival (Table II). Lymph node status, tumour size, S-phase fraction and PR content were independent prognostic factors. In addition, univariate analysis showed that decreasing age, DNA aneuploidy and a low ER content were associated with a high risk of recurrence. Estrogen receptor content became an independent variable if PR status was excluded from the multivariate analysis ($P = 0.010$). Patients with tumours having a low or intermediate SPF had approximately the same risk of recurrence while a S-phase fraction of 10% or greater was associated with a much higher risk. SPF was related to recurrence-free survival in different subgroups of lymph node status. Amongst node-negative patients, two of 118 with a low SPF (<10%) relapsed, while 22 of 78 patients with a high SPF developed metastasis. DNA aneuploid tumours were more aggressive than euploid tumours, especially the subgroup comprising DNA hypodiploid, hypertetraploid and multiploid tumours. This was most evident in the node-negative subgroup ($P < 0.001$). The difference was less pronounced amongst node-positive patients and did not reach statistical significance.

### *Systemically untreated patients*

The distant recurrence-free survival amongst the 212 patients who had not received adjuvant systemic treatment was analysed separately (Table III). Nodal status, S-phase fraction and PR status were identified as independent prognostic factors. Tumours larger than 20 mm were associated with a worse prognosis than smaller tumours, but the multivariate

**Table I** Interrelationships between receptor status, DNA ploidy, S-phase fraction, lymph node status, tumour size and age

| | No. | Receptor status % ER+ | % PR+ | DNA ploidy % Aneuploid* | S-phase fraction Mean (%) |
|---|---|---|---|---|---|
| Lymph node status | | | | | |
| 0 | 196 | 65 | 61 | 49[a] | 9.7[a] |
| 1–3 | 94 | 74 | 64 | 54 | 10.5 |
| >3 | 46 | 52 | 52 | 70 | 11.8 |
| Tumour size | | | | | |
| ⩽20 mm | 192 | 72[b] | 66[a] | 44[c] | 9.0[c] |
| >20 mm | 144 | 58 | 53 | 65 | 11.7 |
| Age | | | | | |
| ⩽40 years | 83 | 58 | 52 | 69[b] | 12.9[c] |
| 41–49 years | 253 | 69 | 63 | 48 | 9.3 |
| ER status | | | | | |
| ER-positive | 222 | | 85[c] | 44[c] | 8.3[c] |
| ER-negative | 114 | | 12 | 71 | 13.8 |
| PR status | | | | | |
| PR-positive | 203 | 93[c] | | 46[b] | 8.4[c] |
| PR-negative | 133 | 25 | | 64 | 12.9 |
| DNA ploidy | | | | | |
| Euploid | 157 | 79[c] | 69[b] | | 6.8[c] |
| Aneuploid | 179 | 55 | 53 | | 13.1 |

*Not including DNA tetraploid tumours. [a]$P < 0.05$, [b]$P < 0.01$, [c]$P < 0.001$.

**Table II** Distance recurrence-free survival analysed by the Cox model for all 336 patients

| | No. of patients | No. of recurrences | Univariate Rate ratio | Univariate Test for trend | Multivariate Adjusted rate ratio | Multivariate Test for trend |
|---|---|---|---|---|---|---|
| Lymph node status | | | | | | |
| 0 | 196 | 24 | 1.0 | | 1.0 | |
| 1–3 | 94 | 19 | 1.6 | $P < 0.001$ | 1.3 | $P < 0.001$ |
| >3 | 46 | 22 | 5.2 | | 4.4 | |
| Tumour size | | | | | | |
| ⩽ 20 mm | 192 | 20 | 1.0 | | 1.0 | |
| > 20 mm | 144 | 45 | 3.2 | $P < 0.001$ | 2.2 | $P = 0.0060$ |
| DNA ploidy | | | | | | |
| Euploid | 157 | 17 | 1.0 | | | |
| Hyperdiploid | 134 | 33 | 2.5 | $P < 0.001$ | | |
| Other aneuploid | 45 | 15 | 3.9 | | | |
| S-phase fraction | | | | | | |
| <5% | 79 | 4 | 1.0 | | 1.0 | |
| 5–10% | 102 | 7 | 1.2 | $P < 0.001$ | 1.1 | $P < 0.001$ |
| ⩾ 10% | 155 | 54 | 7.3 | | 5.6 | |
| ER (fmol $\mu g^{-1}$ DNA) | | | | | | |
| <0.1 | 114 | 38 | 1.00 | | | |
| ⩾ 0.1 | 222 | 27 | 0.33 | $P < 0.001$ | | |
| PR (fmol $\mu g^{-1}$DNA) | | | | | | |
| <0.1 | 133 | 43 | 1.00 | | 1.00 | |
| ⩾ 0.1 | 203 | 22 | 0.34 | $P < 0.001$ | 0.44 | $P = 0.0023$ |
| Age | | | | | | |
| ⩽ 40 years | 83 | 24 | 1.00 | | | |
| > 40 years | 253 | 41 | 0.52 | $P = 0.011$ | | |

analysis showed no significant difference in recurrence rate, with a rate ratio of 1.2 (0.6–2.7, 95% confidence interval). Progesterone receptor status could be replaced by ER status as an independent prognostic variable ($P = 0.013$).

For the systemically untreated patients with node-negative breast cancer, S-phase fraction and PR status were used in combination in order to find groups at low and high risk of recurrence. Approximately two thirds of the patients had tumours with S-phase levels below 10% and showed a low rate of recurrence (Figure 1). Amongst those with a high S-phase fraction, tumours positive for PR seemed to be less aggressive than PR-negative tumours. Tumour size, however, did not contribute prognostic information in addition to S phase fraction.

Amongst node-positive patients who did not receive systemic therapy, cases which were both PR-positive and of low S-phase rate formed a group at low risk comprising one third of the patients (Figure 2). The cumulative recurrence-free survival at 4 years was 90%. As observed in the node-negative subgroup, the risk of relapse for patients with a high SPF was dependent on PR status.

*Local recurrence and breast cancer mortality*

For breast cancer mortality, among the whole patient population, the same independent variables were identified by Cox analysis as were obtained for distant metastasis (Table II). In the node-negative group, none of the 118 patients with a S-phase value below 10% died during the observation period, while the cumulative survival at 4 years was 70% for those having a SPF of 10% or greater. Fourteen patients developed a local recurrence. Those with large tumour size ($P = 0.04$) and those with receptor negative tumours (ER, $P = 0.027$; PR, $P = 0.016$) more often had a local recurrence. Neither lymph node status nor S-phase fraction were significantly related to local recurrence.

**Figure 1** The distant recurrence-free survival related to SPF and PR status in systemically untreated patients with node-negative breast cancer.

**Figure 2** The distant recurrence-free survival related to SPF and PR status in systemically untreated patients with node-positive breast cancer.

### Discussion

S-phase fraction and hormone receptor status contributed prognostic information in addition to lymph node status and tumour size in the present study of young patients. The same has been found in several studies including breast cancer patients of mixed ages (Hatschek *et al.*, 1989; Kallioniemi *et al.*, 1988; Meyer & Province, 1988; Sigurdsson *et al.*, 1990; Stål *et al.*, 1989). This similarity is in line with the fact that age seems not to be an independent prognostic factor in breast cancer (Clark *et al.*, 1989; Kallioniemi *et al.*, 1988; Sigurdsson *et al.*, 1990; Stål *et al.*, 1991), although young patients treated by surgery alone tend to relapse at a higher rate than older ones (Simpson *et al.*, 1988). This probably reflects the fact that young patients more often have tumours which are receptor negative, DNA aneuploid or of high S-phase fraction than have older patients (Olsson *et al.*, 1991; Sigurdsson *et al.*, 1990; Simpson *et al.*, 1988; von Rosen *et al.*, 1986; Wilking *et al.*, 1989). The present series indicates that SPF is the one most strongly related to age. In a recently published study of premenopausal women with breast cancer, a high SPF in the tumour was related to early use of oral contraceptives (Olsson *et al.*, 1991).

While the clinical significance of ER and PR status is well established in subgroups of patients receiving adjuvant hormonal treatment, the prognostic value in node-negative patients treated by surgery alone, or in combination with radiotherapy, is more controversial. For the latter subgroup, premenopausal patients with receptor negative tumours relapsed at a higher rate than those with receptor positive tumours in two studies (Moot *et al.*, 1987; Thorpe *et al.*, 1987), while there was no survival difference due to receptor status amongst postmenopausal women in one of the studies (Thorpe *et al.*, 1987). On the other hand, a better prognosis for patients with ER-positive tumours in the postmenopausal group, but not in the premenopausal one was reported by Crowe and colleagues (1982). Amongst the 169 systemically untreated node-negative patients in the present study, PR status was related to recurrence-free survival in the subgroup with a high SPF (Figure 1). Furthermore, if all systemically untreated patients were considered, PR status contributed prognostic information in addition to that of nodal status and SPF (Table III). To our knowledge, this relationship has not been observed before.

Does a low SPF alone identify node-negative patients at low risk? In other studies of early breast cancer, tumour size has been taken into account in addition to SPF (O'Reilly *et al.*, 1990*a*; Sigurdsson *et al.*, 1990). In the present study of young patients, however, tumour size showed no additional value, which may be due to its correlation with SPF (Table I). The relatively high correlation between tumour size and SPF may in turn be the result of repeatedly mammography screening. Our data suggest that the subgroup of patients with a high SPF could be candidates for adjuvant therapy. For receptor positive cases, tamoxifen treatment has shown to be effective in premenopausal as well as postmenopausal patients (Fisher *et al.*, 1989).

Amongst node-positive patients, PR-positive tumours with low SPF formed a subgroup at low risk, comprising one third of the patients (Figure 2). In contrast to our study, untreated node-positive patients with low levels of SPF showed fairly poor prognosis in the study of O'Reilly *et al.* (1990*b*) with a 3-year recurrence-free survival rate of approximately 50%. A possible explanation for this difference may be, that while small tumours were rare in the English study, almost half of the node-positive cases in the present series were sized 20 mm or less. Possibly, the relatively large proportion of patients identified at low risk in the present study was influenced by population screening. Of the 15 untreated patients identified at low risk, only two showed involvement in more than three lymph nodes. Thus, the low-risk group may not be representative for those having more than three positive nodes, who were associated with a very poor prognosis (Table III).

The prognostic importance of ER content was close to that

**Table III** Multivariate analysis of the distant recurrence-free survival amongst systemically untreated patients (Cox model)

| | *No. of patients* | *No. of recurrences* | *Adjusted rate ratio* | *Test for trend* |
|---|---|---|---|---|
| Lymph node status | | | | |
| 0 | 169 | 15 | 1.0 | |
| 1–3 | 29 | 7 | 2.7 | $P<0.001$ |
| >3 | 14 | 9 | 14.5 | |
| S-phase fraction | | | | |
| <5% | 53 | 1 | 1.0 | |
| 5–10% | 75 | 4 | 1.9 | $P<0.001$ |
| ⩾10% | 84 | 26 | 10.3 | |
| PR (fmol $\mu g^{-1}$ DNA) | | | | |
| <0.1 | 79 | 22 | 1.00 | |
| ⩾0.1 | 133 | 9 | 0.31 | $P=0.0046$ |

of PR content in the present study, which is in agreement with the high correlation observed between the two variables. For postmenopausal patients the correlation has been shown to be less close due to a relatively higher proportion of ER + PR – tumours in this subgroup (Thorpe, 1987; Wilking *et al.*, 1989).

As in the present study, DNA ploidy has shown prognostic significance in node-negative breast cancer in contrast to node-positive in some studies (Ewers *et al.*, 1989; Toikkanen *et al.*, 1989). In the series of node-positive breast cancer reported by Baildam *et al.* (1987), Hedley *et al.* (1987) and Lykkesfeldt *et al.* (1988), DNA ploidy predicted recurrence or mortality, but not independent of other prognostic factors. No difference in recurrence-free survival due to DNA ploidy was observed amongst premenopausal node-positive patients in the study of Cornelisse *et al.* (1987). In contrast, SPF appears to be an independent prognostic factor in both subgroups of lymph node status, even after long follow-up periods (Toikkanen *et al.*, 1989). However, as SPF in most studies is not assessed in approximately 20% of the samples, DNA ploidy should be useful in such cases, especially as regards node-negative breast cancer. In the present series, the subgroup with DNA hypodiploid, hypertetraploid or multiploid tumours exhibited the highest recurrence rate. In patients aged 40 years or less, 11% of the tumours were DNA hypodiploid, compared with 5% amongst those between 41 and 49 years of age. This frequency continues to decrease with increasing age (Stål *et al.*, 1992). Further investigations are needed to explain this relationship.

While a high SPF strongly correlated with distant recurrence in the present study, it did not significantly predict local recurrence. Similar results have been obtained by others (Héry *et al.*, 1987; Hatschek *et al.*, 1989). Probably, frequency of local recurrence is most influenced by the type of local treatment, such as radiotherapy.

In conclusion, flow-cytometric S-phase fraction and hormone receptor status have clinical significance in breast cancer patients aged less than 50 years. A high SPF identified a subgroup with high recurrence rate, comprising approximately one third of the node-negative patients. Similarly, the one third of node-positive patients who had PR-positive tumours with a low S-phase fraction formed a subgroup with a low recurrence rate.

### References

ARMITAGE, P. & BERRY, G. (1987). *Statistical Methods in Medical Research*. Blackwell Scientific Publications: Oxford.

BAILDAM, A.D., ZALOUDIK, J., HOWELL, A. & 5 others (1987). DNA analysis by flow cytometry, response to endocrine treatment and prognosis in advanced carcinoma of the breast. *Br. J. Cancer*, **55**, 553.

BAISCH, H., GÖHDE, W. & LINDEN, W.A. (1975). Analysis of PCP-data to determine the fraction of cells in the various phases of the cell cycle. *Radiat. Environ. Biophys.*, **12**, 31.

CARTER, C.L., ALLEN, C. & HENSON, D.E. (1989). Relation of tumor size, lymph node status, and survival in 24,740 breast cancer cases. *Cancer*, **63**, 181.

CLARK, G.M., DRESSLER, L.G., OWENS, M.A., POUNDS, G., OLDAKER, T. & MCGUIRE, W.L. (1989). Prediction of relapse or survival in patients with node-negative breast cancer by DNA flow cytometry. *N. Engl. J. Med.*, **320**, 627.

CORNELISSE, C.J., VAN DE VELDE, C.J.H., CASPERS, R.J.C., MOOLENAAR, A.J. & HERMANS, J. (1987). DNA ploidy and survival in breast cancer patients. *Cytometry*, **8**, 225.

COX, D.R. (1972). Regression models and life tables (with discussion). *J. Stat. Soc. B*, **34**, 187.

CROWE, J.P., HUBAY, C.A., PEARSON, O.H. & 7 others (1982). Estrogen receptor status as a prognostic indicator for stage I breast cancer patients. *Breast Cancer Res. Treat.*, **2**, 171.

EARLY BREAST CANCER TRIALISTS' COLLABORATIVE GROUP (1988). Effects of adjuvant tamoxifen and of cytotoxic therapy on mortality in early breast cancer: An overview of 61 randomized trials among 28,896 women. *N. Engl. J. Med.*, **319**, 1681.

EWERS, S.B., BALDETORP, B., KILLANDER, D. & LÅNGSTRÖM, E. (1989). Flow cytometry, DNA ploidy and number of cell populations in the primary breast cancer and their correlation to the prognosis. *Acta Oncol.*, **28**, 913.

FERNÖ, M., BORG, Å. & SELLBERG, G. (1986). Enzyme immuno assay of the estrogen receptor in breast cancer biopsy samples. A comparison with isoelectric focusing. *Acta. Radiol. Oncol.*, **25**, 171.

FISHER, B., CONSTANTINO, J., REDMOND, P.H.C. & 17 others (1989). A randomized clinical trial evaluating tamoxifen in the treatment of patients with node-negative breast cancer who have estrogen-receptor-positive tumors. *N. Engl. J. Med.*, **320**, 479.

GODOLPHIN, W., ELWOOD, J.M. & SPINELLI, J.J. (1981). Estrogen receptor quantitation and staging as complementary prognostic indicators in breast cancer: a study of 583 patients. *Int. J. Cancer*, **28**, 677.

HATSCHEK, T., FAGERBERG, G., STÅL, O. & 4 others (1989). Cytometric characterization and clinical course of breast cancer diagnosed in a population based screening program. *Cancer*, **64**, 1074.

HEDLEY, D.W., RUGG, C.A. & GELBER, R.D. (1987). Association of DNA index and S-phase fraction with prognosis of nodes positive early breast cancer. *Cancer Res.*, **47**, 4729.

HÉRY, M., GIOANNI, J., LALANNE, C.-M., NAMER, M. & COURDI, A. (1987). The DNA labelling index: a prognostic factor in node-negative breast cancer. *Breast Cancer Res. Treat.*, **9**, 207.

KALLIONIEMI, O.-P., BLANCO, G., ALAVAIKKO, M. & 5 others (1988). Improving the prognostic value of DNA flow cytometry in breast cancer by combining DNA index and S-phase fraction. *Cancer*, **62**, 2183.

KAPLAN, E. & MEIER, P. (1958). Nonparametric estimation from incomplete observations. *J. Amer. Statist. Assoc.*, **53**, 457.

LYKKESFELDT, A.E., BALSLEV, I., CHRISTENSEN, I.J. & 5 others (1988). DNA ploidy and S-phase fraction in primary breast carcinomas in relation to prognostic factors and survival for premenopausal patients at high risk for recurrent disease. *Acta Oncol.*, **27**, 749.

MEYER, J.S. & PROVINCE, M. (1988). Proliferative index of breast carcinoma by thymidine labeling: prognostic power independent of stage, estrogen and progesterone receptors. *Breast Cancer Res. Treat.*, **12**, 191.

MOOT, S.K., PETER, G.N. & CHEEK, J.H. (1987). Tumor hormone receptor status and recurrences in premenopausal node negative breast carcinoma. *Cancer*, **60**, 382.

OLSSON, J., RANSTAM, J., BELDETORP, B. & 4 others (1991). Proliferation and DNA ploidy in malignant breast tumors in relation to early oral contraceptive use and early abortions. *Cancer*, **67**, 1285.

O'REILLY, S.M., CAMPLEJOHN, R.S., BARNES, D.M., MILLIS, R.R., RUBENS, R.D. & RICHARDS, M.A. (1990*a*). Node-negative breast cancer: Prognostic subgroups defined by tumor size and flow cytometry. *J. Clin. Oncol.*, **8**, 2040.

O'REILLY, S.M., CAMPLEJOHN, R.S., MILLIS, R.R., RUBENS, R.D. & RICHARDS, M.A. (1990*b*). Proliferative activity, histological grade and benefit from adjuvant chemotherapy in node positive breast cancer. *Eur. J. Cancer*, **26**, 1035.

SIGURDSSON, H., BALDETORP, B., BORG, Å. & 4 others (1990). Indicators of prognosis in node-negative breast cancer. *N. Engl. J. Med.*, **322**, 1045.

SILVESTRINI, R., DAIDONE, M.G., VALAGUSSA, P., DI FRONZO, G., MEZZANOTTE, G. & BONADONNA, G. (1989). Cell kinetics as a prognostic indicator in node-negative breast cancer. *Eur. J. Clin. Oncol.*, **25**, 1165.

SIMPSON, H.W., PAUSON, A.W., GRIFFITHS, K., CANDLISH, W., MCARDLE, C.S. & SMALL, R.G. (1988). Genesis of breast cancer is in the premenopause. *Lancet*, **ii**, 74.

SKOOG, L., HUMLA, S., AXELSSON, M. & 4 others (1987). Estrogen receptor levels and survival of breast cancer patients. A study on patients participating in randomized trials of adjuvant therapy. *Acta Oncol.*, **26**, 95.

SPYRATOS, F., HACENE, K., TUBIANA-HULIN, M., PALLUD, C. & BRUNET, M. (1989). Prognostic value of estrogen and progesterone receptors in primary infiltrating ductal breast cancer. A sequential multivariate analysis of 1262 patients. *Eur. J. Cancer Clin. Oncol.*, **25**, 1233.

STÅL, O., BRISFORS, A., CARSTENSEN, J., FERRAUD, L., HATSCHEK, T. & NORDENSUJÖLD, B. (1992). Interrelations between cellular DNA content, S-phase fraction, hormone receptor status and age in primary breast cancer. A series of 1342 consecutively detected tumors. *Acta Oncol.*, **31**, 283.

STÅL, O., HATSCHEK, T., CARSTENSEN, J. & NORDENSKJÖLD, B. (1991). DNA analysis in the management of breast cancer. *Diagn. Oncol.*, **1**, 140.

STÅL, O., WINGREN, S., CARSTENSEN, J. & 4 others (1989). Prognostic value of DNA ploidy and S-phase fraction in relation to estrogen receptor content and clinicopathological variables in primary breast cancer. *Eur. J. Cancer Clin. Oncol.*, **25**, 301.

THORPE, S.M. (1987). Immunological quantitation of nuclear receptors in human breast cancer: relation to cytosolic estrogen and progesterone receptors. *Cancer Res.*, **47**, 1830.

THORPE, S.M., ROSE, C., RASMUSSEN, B.B., MOURIDSEN, H.T., BAYER, T. & KEIDING, N. (1987). Prognostic value of steroid hormone receptors: multivariate analysis of systemically untreated patients with node negative primary breast cancer. *Cancer Res.*, **47**, 6126.

TOIKKANEN, S., JOENSUU, H. & KLEMI, P. (1989). The prognostic significance of nuclear DNA content in invasive breast cancer - a study with long-term follow-up. *Br. J. Cancer*, **60**, 693.

VINDELÖV, L.L., CHRISTENSEN, I.J. & NISSEN, N.I. (1983). A detergent-trypsin method for the preparation of nuclei for flow cytometric DNA analysis. *Cytometry*, **3**, 323.

WILKING, N., RUTQVIST, L.E., NORDENSKJÖLD, B. & SKOOG, L. (1989). Steroid receptor levels in breast cancer. Relationships with age and menopausal status. *Acta. Oncol.*, **28**, 807.

VON ROSEN, A., FALLENIUS, A., SUNDELIN, B. & AUER, G. (1986). Nuclear DNA content in mammary carcinoma in women aged 35 or younger. *Am. J. Clin. Oncol.*, **9**, 382.

WRANGE, Ö., NORDENSKJÖLD, B. & GUSTAFSSON, J.-Å. (1978). Cytosol estradiol receptor in human mammary carcinoma: an assay on isoelectric focusing in polyacrylamide gel. *Anal. Biochem.*, **85**, 461.